# Impact of side-hole geometry on the performance of hemodialysis catheter tips: A computational fluid dynamics assessment

**DOI:** 10.1371/journal.pone.0236946

**Published:** 2020-08-07

**Authors:** David G. Owen, Diana C. de Oliveira, Shuang Qian, Naomi C. Green, Duncan E. T. Shepherd, Daniel M. Espino

**Affiliations:** Department of Mechanical Engineering, University of Birmingham, Birmingham, United Kingdom; University of New South Wales, AUSTRALIA

## Abstract

Hemodialysis catheters are used to support blood filtration, yet there are multiple fundamentally different approaches to catheter tip design with no clear optimal solution. Side-holes have been shown to increase flow rates and decrease recirculation but have been associated with clotting/increased infection rates. This study investigates the impact of changing the shape, size and number of side-holes on a simple symmetric tip catheter by evaluating the velocity, shear stress and shear rate of inflowing blood. A platelet model is used to examine the residence time and shear history of inflowing platelets. The results show that side-holes improve the theoretical performance of the catheters, reducing the maximum velocity and shear stress occurring at the tip compared to non-side-hole catheters. Increasing the side-hole area improved performance up to a point, past which not all inflow through the hole was captured, and instead a small fraction slowly ‘washed-out’ through the remainder of the tip resulting in greater residence times and increasing the likelihood of platelet adhesion. An oval shaped hole presents a lower chance of external fibrin formation compared to a circular hole, although this would also be influenced by the catheter material surface topology which is dependent on the manufacturing process. Overall, whilst side-holes may be associated with increased clotting and infection, this can be reduced when side-hole geometry is correctly implemented though; a sufficient area for body diameter (minimising residence time) and utilising angle-cut, oval shaped holes (reducing shear stress and chances of fibrin formation partially occluding holes).

## Introduction

Hemodialysis is the process by which blood is removed via a dialysis catheter situated in the right atrium or superior vena cava (SVC), filtered externally, then resupplied back into the bloodstream through the same catheter. Chronic kidney disease (CKD) affects an estimated 15% of adults in the USA [1], with more than 726,000 receiving dialysis [2]. As patients typically receive 15 hours of hemodialysis per week [3], the optimal and efficient function of hemodialysis catheters is important for expedited blood filtration, with a wide variety of tip designs and features attempting to achieve this.

Typical problems affecting catheter performance are recirculation of dialysed blood [4], bio-film and fibrin formation [5], shear induced platelet activation [6] and regions of flow stagnation [7]. One of the current leading tip designs is widely considered to be a symmetric style tip [4, 8]; however, there are multiple different design concepts including the Palindrome (Medtronic, Dublin, Ireland), Vector-Flow (Tele-Flex, Pennsylvania, United Sates) and Glide-path (CR-Bard, New Jersey, United States). These designs include a variety of features with different geometry side-holes (circular, oval, trapezoidal) located in a variety of configurations [8].

Comparisons of catheter performance have been performed both *in vitro* [8, 9] and *in silico* [10, 11] to identify favourable design features; however, due to the large variability in design between catheters, it is difficult to conclude if a design is optimal or how different features are impacting the flow into the catheter. In particular, whilst side-holes improve flow rates [12] and reduce recirculation [11], they have been associated with elevated susceptibility to clot formation and anchoring, increased rates of infection and leakage of heparin solution [12, 13]. A key factor in the design of catheter tips is the reduction of shear stress during inflow as elevated shear stress levels >10 Pa being a potential threshold for platelet activation and blood damage [14]. Therefore, careful evaluation of side-hole performance must be performed before incorporation into any design.

This study aims to investigate the impact of different side-hole configurations on the local hemodynamics and catheter performance through several computational fluid dynamic (CFD) studies. The variation of size, shape and number of side-holes will be assessed by comparing velocities and flow rates occurring at the tip, regions of high shear stress during inflow, and the effect on platelets, including their path-lines, residence time and shear history.

## Methods

### Catheter designs

#### Base symmetric design

**Fig 1:**
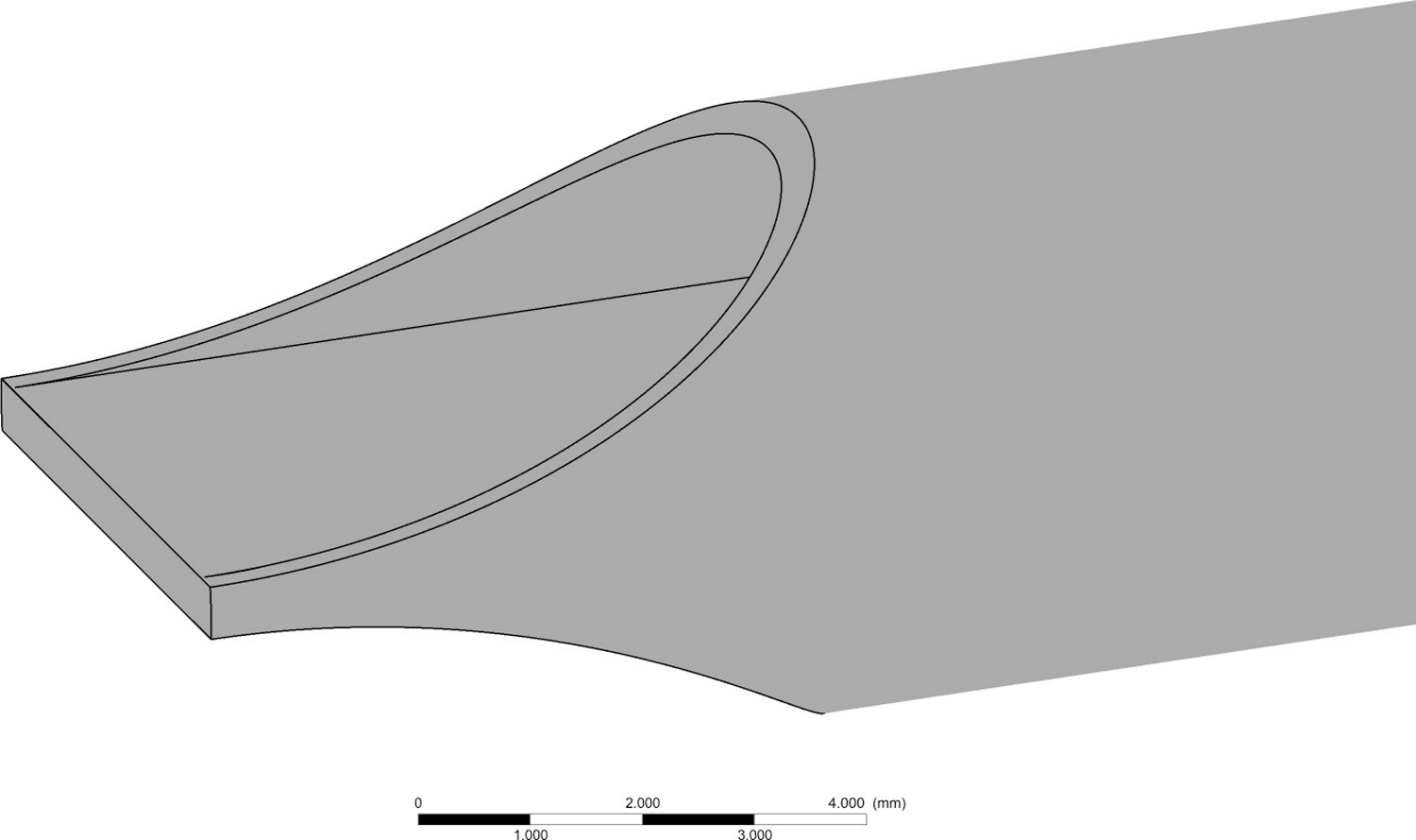
Base symmetric tip design.

The catheter design used for this analysis is similar to many commercially available designs: a generic, symmetric style tip with two semi-circular lumens (5 mm internal diameter) and an outer body diameter of 5.5 mm (16.5 Fr) [8]. All geometry was created using Ansys DeisgnModeller (Ansys Inc, Pennsylvania, United states). Catheter lumens are typically manufactured from extruded polyurethane, with tip features applied using drilling, skive tools and bonding [15]. The tip opening is achieved with a 10 mm radius skive cutting tool, resulting in an ‘angled-cut’ profile and leaving a 0.5 mm septum separating the two lumens as shown in Fig 1.

#### Variations in design

**Fig 2:**
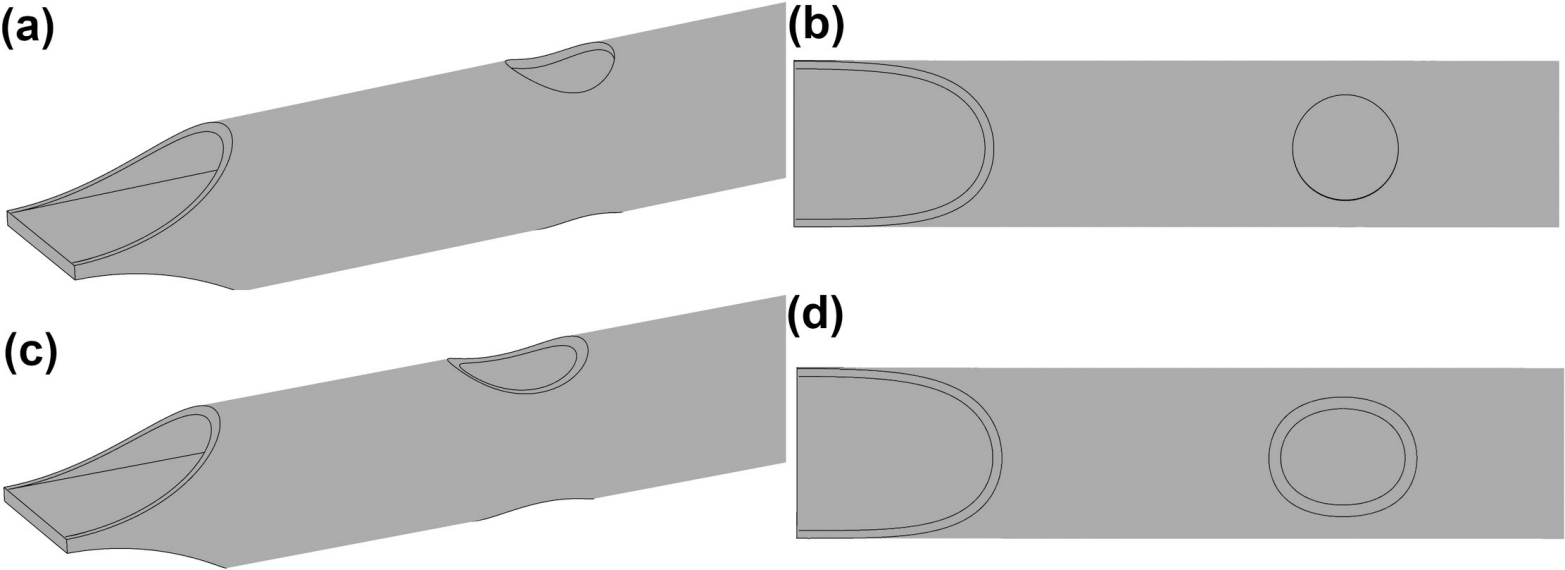
Medium size side-holes for the circular (a)-(b) and oval (c)-(d) shaped catheters.

The two shapes of side-hole considered are; a simple circular hole which could be drilled or punched into the catheter body resulting in a ‘straight-cut’ profile (Fig 2A), and a rounded oval shape achieved using the same skive tool for the tip opening resulting in a ‘angled-cut’ profile (Fig 2C). The size of these holes is reported in Table 1, with each hole positioned such that the smallest distance between the end of the hole and end of the tip is constant (16.5 mm) in all designs.

**Table 1:** Areas of the small, medium and large side-holes.

Shape	Small Area (mm^2^)	Medium Area (mm^2^)	Large Area (mm^2^)
Circular	7.06	9.62	12.57
Oval			

**Fig 3:**
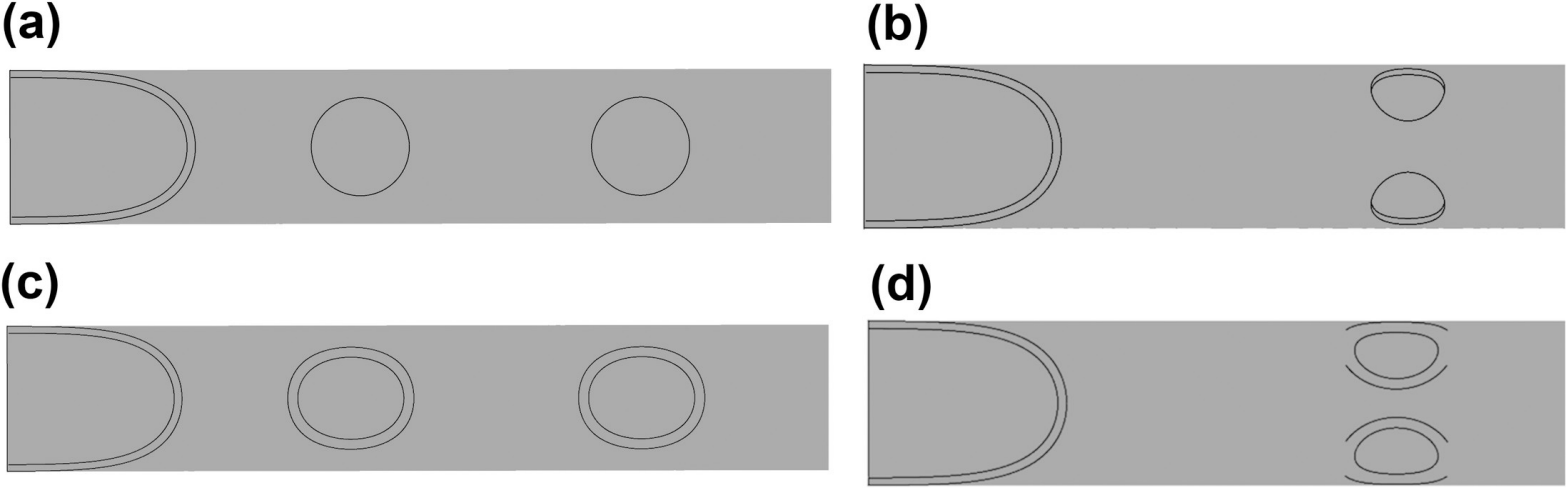
Linear (a,c) and Parallel (b,d) dual-hole configurations for the circular and oval holes.

After analysing the impact of the shapes/areas described above on catheter performance, the best performing area will then be split into two equally sized holes and evaluated in a dual-hole configuration as show in Fig 3. Dual-hole configurations are employed in multiple tip designs [8], such as Equistream (CR-Bard, New Jersey, United States), Hemosplit XK (CR-Bard, New Jersey, United States) and the Ash Split (Medcomp, Pennsylvania, United States), as they can distribute flow evenly and reduce the impact on performance if one hole becomes occluded.

### CFD model

#### Model domain

**Fig 4:**
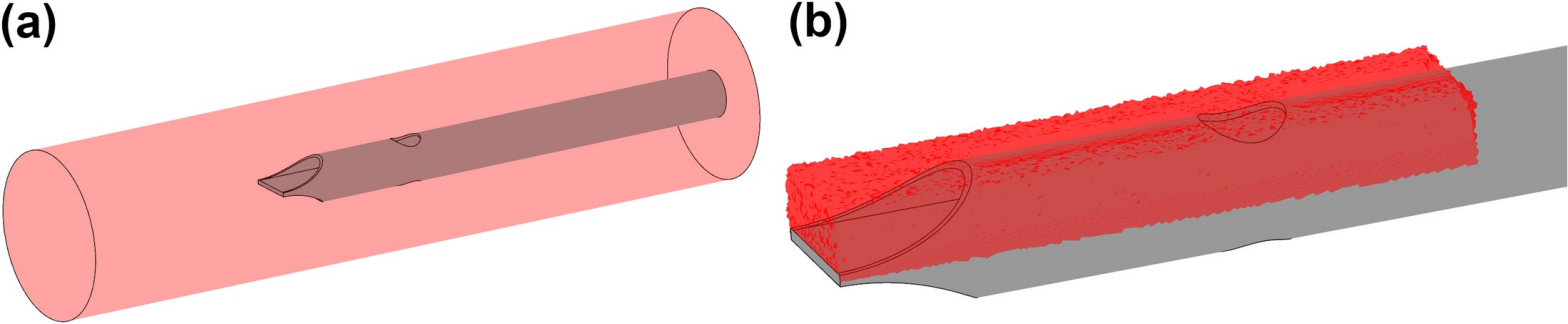
(a) SVC enclosure showing catheter placement, (b) Tip volume for medium sized circular hole.

Similar to other CFD catheter studies [10, 11, 16], the catheters are enclosed in a 20 mm diameter cylinder representing the superior vena cava (SVC) to allow for uniform comparisons on performance. The SVC model is 90 mm long, with a 60 mm length catheter inserted to allow sufficient distance for fully normalised inflow to the catheter to develop and no outlet effects to occur (Fig 4A). To compare inflow parameters across multiple designs, a cuboid ‘tip-volume’ is defined for the inflow lumen of each catheter design (Fig 4B) with an average volume of 350 ml. The tip volume has a width of 5 mm and a height of 2.5 mm to include the entire inflow lumen. It extends from the most distal point of the catheter up to a plane proximal to the side-hole where the inflow velocity has become stable/fully developed, with no further flow disturbances occurring until the blood exits the catheter base. This focuses the comparisons between the different configurations on the most critical regions of inflowing blood.

#### Mesh convergence

**Fig 5:**
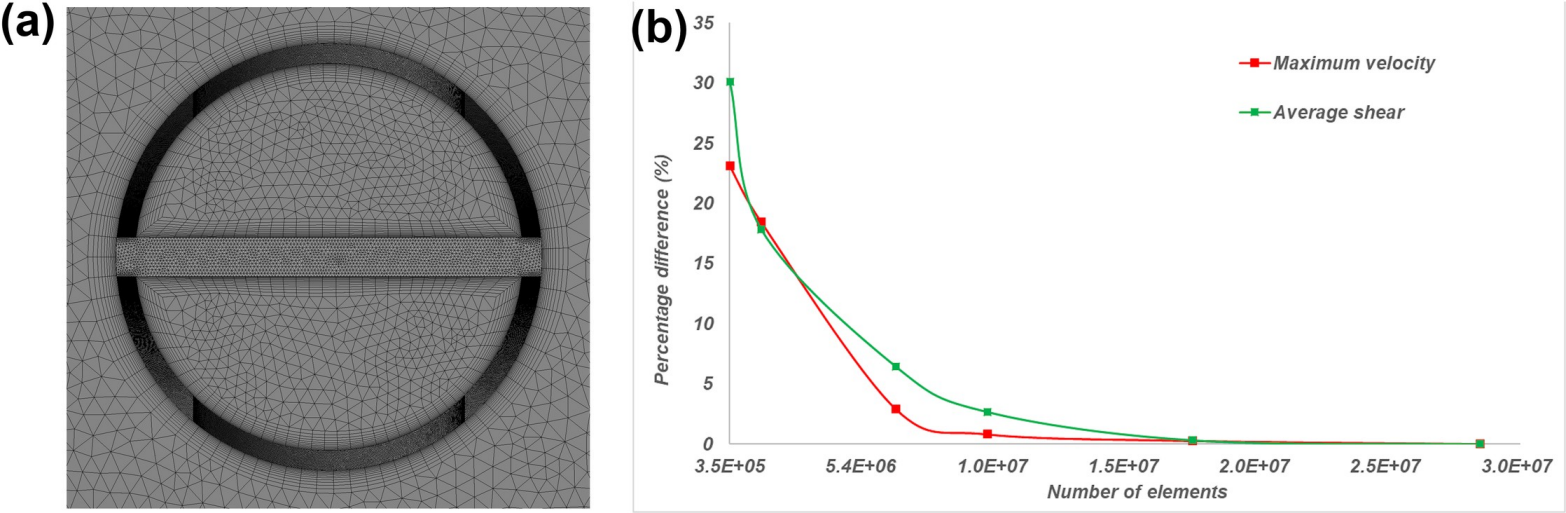
(a) Cross section of mesh, (b) Percentage difference with increasing elements for maximum tip velocity and average shear rate in tip.

Simulations were performed on the medium sized circular hole tip design at six incrementally increasing mesh densities. The mesh used in the study was chosen once the percentage difference for maximum velocity and average shear rate in the tip was below 0.5%. This resulted in a 18,000,165 element predominantly tetrahedral mesh, with 12 prismatic layers surrounding the inner/outer lumens of the catheter body. The average skewness and orthogonal quality of the mesh was 0.2 and 0.77 respectively [17], with a cross section of the mesh shown in Fig 5A and the percentage differences for the varying mesh densities in Fig 5B.

#### Blood model

Blood is a non-Newtonian fluid [18], which exhibits shear-thinning behaviours at higher shear rates [19] (>100 s^-1^). Blood can often be assumed Newtonian in specific physiological applications [20], however, given the range of shear rates that occur during inflow a suitable non-Newtonian blood model should be selected. This study uses an asymptotic shear-thinning Carreau model [21] which uses the definition of shear rate in Eq (1) and is defined in Eq (2).

$$\dot{\gamma }=\sqrt{2*D_{ij}\cdot D_{ij}}$$ (1)

where $\dot{\gamma }$ is the shear rate of the fluid, *D* is the strain rate tensor with *i*, *j* = 1,2,3 as the inner products.

$$\mu =\mu_{\infty }+\left(\mu_{\infty }+\mu_{0}\right){\left[1+{\left(\lambda \dot{\gamma }\right)}^{2}\right]}^{\frac{n-1}{2}}$$ (2)

Where *μ* is the viscosity of blood and *μ*_∞_ = 0.00345 *Pa*. *s*, *n* = 0.25, *μ*_0_ = 0.025 *Pa*. *S*, *λ* = 25 *s*.

#### Platelet modelling

To further analyse the different designs, the path lines of platelets flowing through the side-holes is also considered. Platelets are generated on a tangential plane 1 mm above the surface of the catheter and are tracked through the flow field across steps of 0.5 m. Platelets are modelled as rigid spheres with a diameter [22] of 2 μm which experience drag modelled by the Schiller-Naumann model [23] (Eq 3) and lift due to shear via the Saffman lift model [24] (Eq 4). Whilst this is the first use of this model applied to platelets, it has been applied to other blood consitiuents and lung particulates with a review of implentations by Kleinstreuer and Feng [25].

$$C_{D}=\{\begin{array}{c} \frac{24\left(1+0.15Re^{0.687}\right)}{Re}\;if\;Re\leq 1000\\ 0.44\;if\;Re>1000 \end{array}$$ (3)

$$\vec{F_{L}}=\frac{2Kv^{\frac{1}{2}}d_{ij}}{d_{p}\left(d_{lk}d_{kl}\right)}\left(\vec{u}-\vec{u_{p}}\right)$$ (4)

where *Re* is the Reynolds Number, *K* is a constant equal to 2.594, *v* is the kinematic viscosity, *d*_*i*,*j*_ is the deformation tensor, *d*_*p*_ is the diameter of the platelet, $\vec{u}$ is the velocity of the fluid and $\vec{u_{p}}$ is the velocity of the platelet.

This model allows for the calculation of path lines, shear forces and residence times for each individual platelet during inflow. To assess the potential damage occurring to the platelets, the platelet lysis index (PLI) (Eq 5) is used. This was first introduced by Giersiepen *et al*. [26] for mechanical heart valves and has been previously applied to hemodialysis catheters [10, 16]. The PLI indicates regions where platelets experience higher shear stress and longer residence times (as potential sites for adhesion and thrombus formation) based upon path lines.

$$PLI=At_{p}{}^{0.77}\tau_{p}{}^{3.075}$$ (5)

where *A* is a constant equal to 3.31x10^-6^, *t*_*p*_ is the residence time of the platelet and *τ*_*p*_ is the shear stress acting on the platelet. Contrary to previous studies which used a velocity weighted average [10, 16], in this study a value for the PLI is calculated at each step (0.5 μm) using Eq 5, which is then summated for each line and averaged over all steps to provide an overall PLI magnitude for each catheter to be used for comparisons between designs.

#### Boundary conditions

Blood flow through the SVC was a constant velocity of 0.3 m/s based upon peak values from Mareels *et al*. [16] as transient effects have been shown negligible in other comparative CFD catheter studies [16]. The flow at the catheter inlet was constant at 400 mL/min, with a negative pressure applied at the catheter outlet [7] varied until a flow rate of -400 mL/min was achieved, with the average magnitude across all configurations being -1810 Pa. The walls of the catheter and SVC had a no-slip condition, with the SVC outlet set to a constant gauge pressure of 0 Pa.

#### Solver settings

The governing equations for conservation of mass and momentum for a 3D incompressible fluid were solved numerically using the commercial finite-volume Ansys Fluent v19.2 (Ansys Inc, Pennsylvania, United States) SIMPLE solver. The flow was assumed laminar similar to other comparative CFD catheter studies [10, 11]. Two criteria were used to assess convergence; a mass continuity residual magnitude < 10^−5^ and a outflow rate of 400 ± 1 mL/min. The models were solving using parallel processing on Linux based HPC architecture, with all models utilising 200 cores for 3 hours.

## Results

### Variation in side-hole area

Tip volume results from increasing the area of the side-holes are shown in Table 2, with the inclusion of the same symmetric tip without side-holes (Fig 1) as reference.

**Table 2:** Tip volume parameters for the varying sized circular/oval side-holes.

Tip Design	Side-hole area	Inflow tip volume measurements			
		Max Velocity (m/s)	Average shear stress (Pa)	Percentage Shear stress >10 Pa	Side-hole flow (mL/min)
No hole	-	1.923	5.16	13.75%	-
Circular	Small	1.684	3.70	7.94%	308
	Medium	1.804	3.30	7.53%	363
	Large	1.794	3.30	7.52%	363
Oval	Small	1.866	3.80	8.37%	319
	Medium	1.942	3.43	8.08%	382
	Large	1.982	3.42	7.58%	438

From Table 2, it is clear that the addition of side-holes to catheters can reduce the maximum velocity of inflowing blood (-0.239 m/s for small circular hole), as well as reducing both the average shear stress in the tip and the percentage shear stress above 10 Pa, a potential threshold for platelet activation and blood damage [14].

**Fig 6:**
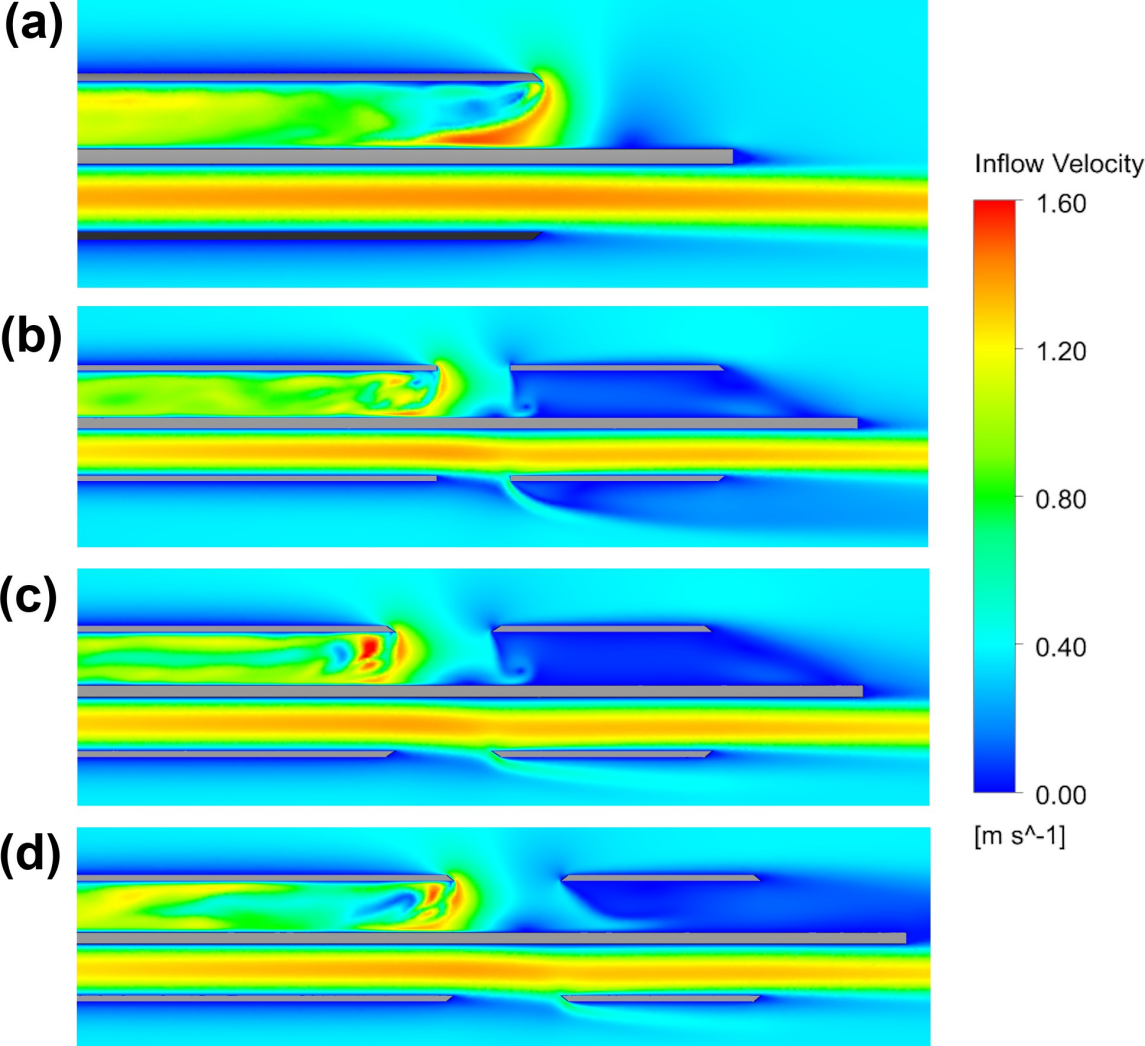
Velocity contours for the no-hole (a), Medium circular (b), Medium Oval (c) and Large oval (d).

It can be observed that side-hole area positively correlates with maximum velocity of blood in the tip and total flow (mL/min) through the hole, but negatively correlates with both of the stress related parameters. However, it should be noted that the increase from small to medium area is much greater than from the medium to large areas. As flow patterns through the side-holes were consistent across almost all configurations, velocity contours along the centreline for a representative sample of the tip designs are shown in Fig 6 with contours of the remainder available in the supplementary data.

Velocity contours for the two medium-sized side-holes are similar; however, the ‘angled-cut’ of the oval hole results in a slightly more unstable flow structure which leads to a higher maximum velocity. The vortex formed by the blood changing direction within the catheter lumen (in the redundant distal section of the tip) was present in all catheter models except for the large oval side-hole (Fig 6D). In the majority of the catheters, the pressure gradient is sufficient to capture all inflow from the side-holes and some smaller additional inflow from the distal tip resulting in a combined flow of 400 mL/min. In the large oval shaped side-hole, this pressure gradient is insufficient to capture all of the flow through the side-hole, and instead the additional 38 mL/min ‘washes-out’ upstream of the side-hole (Fig 6D). This likely occurs as the circular hole concentrates a greater proportion of the inflow at the most proximal point to the base of the catheter compared to the more gradual curvature of the oval shape.

Whilst this study predominantly focuses on the inflow to the catheter as this experiences the most critical flow conditions for both performance and blood damage, the inclusion of side-holes can also be seen to impact the outflow. Comparing outflow from Fig 6A to the other catheter designs, it can be inferred that side-holes increase shear stress of outflowing blood as without side-holes there is zero impedance to the blood exiting the catheter, whereas all designs which include side-holes apply additional shear to this outflowing blood as it is redirected out the side-hole. In particular, the angle of outflow in the ‘straight-cut’ circular side-hole is much greater than the ‘angled-cut’ of the oval shape side-hole which redirects the outflow more gradually.

**Fig 7:**
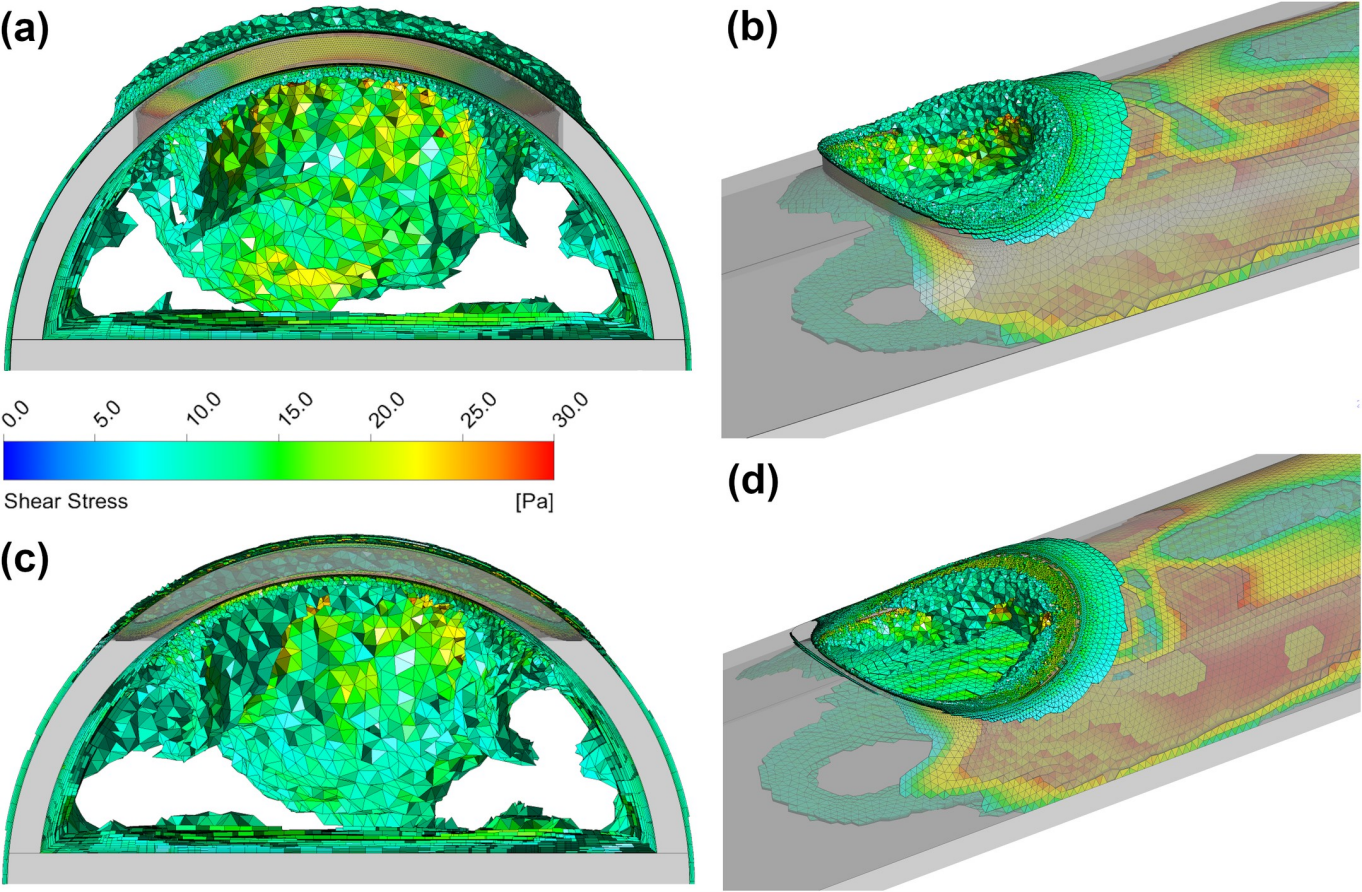
Regions inside the tip volume where blood shear stress is >10 Pa for the medium sized circular (a)-(b) and medium sized oval side-holes (c)-(d).

The contours in Fig 7 show that the main region where shear stress is >10 Pa occurs predominantly at along the centreline of the catheter where the majority of inflow is occurring, hence blood in this region experiences the highest shear rate. This distribution was found across all sizes of side-holes. Both shapes of side-hole have two clear regions where the shear stress is <10 Pa, which occurs in places where blood experiences a more gradual redirection, such as the vortex mentioned previously. Whilst the shape of the side-hole has minimal effect overall on the shear stress within the tip volume (<4% difference in average shear stress for medium size), a much larger region where shear stress in >10 Pa can be seen on the exterior of the circular hole. Results for the simulation of platelet inflow are tabulated in Table 3.

**Table 3:** Platelet parameters for the varying sized circular/oval side-holes.

Tip Design	Side-hole size	Inflow platelet model				
		Average residence time (s)	PLI	Average shear stress (Pa)	Percentage Shear stress >10 Pa	Average shear (s^-1^)
Circular	Small	0.0081	0.0126	8.85	32.04%	2449
	Medium	0.0103	0.0249	8.05	27.23%	2222
	Large	0.0103	0.0505	8.01	26.84%	2212
Oval	Small	0.0085	0.0039	8.82	26.86%	2444
	Medium	0.0110	0.0183	8.62	25.59%	2390
	Large	0.0184	0.0340	7.30	24.88%	2017

**Fig 8:**
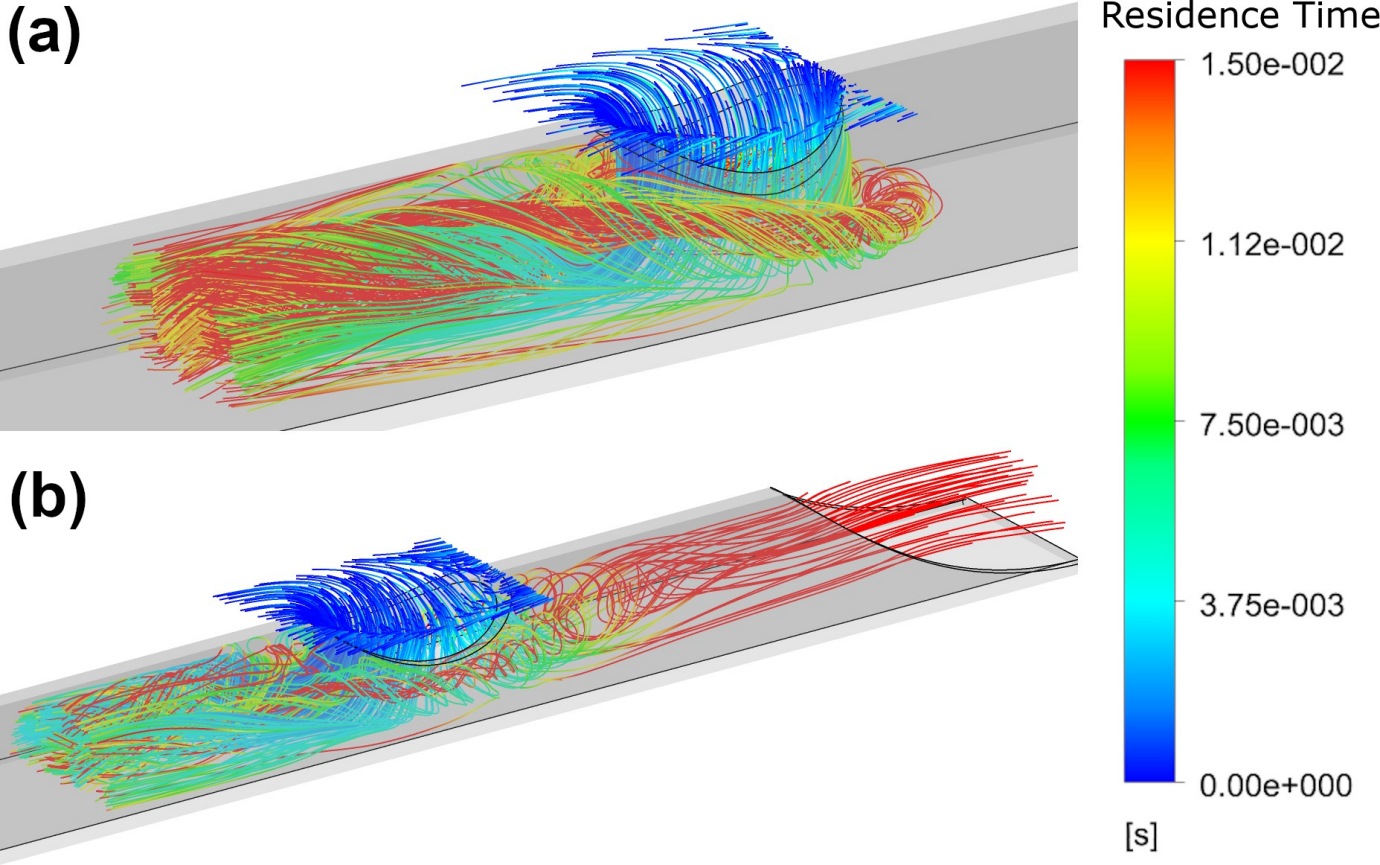
Residence time of platelets for the medium circular (a) and the large oval (b) side-holes.

The results from the platelet modelling are consistent with the tip volume assessment, indicating a reduction in both shear and shear stress acting on the platelets with an increasing side-hole area. Increased side-hole area is shown to increase platelet residence time and also increases the PLI magnitude for both shaped side-holes, with oval shaped holes having the lowest PLI magnitudes. The platelet inflow is visualised in Fig 8.

Whilst the majority of platelet inflow is immediately carried downstream, increasing side-hole area causes increased residence time. Platelets with the greatest residence time can be seen circulating within the lumen (Fig 8A), in regions of low velocity and shear before the pressure gradient is sufficient to remove them downstream. In the case of the large oval-shaped side-hole, some flow leaks through the rest of the tip as not all the flow through the side-hole travels out through the base, resulting in platelet residence times of up to 0.2 s.

### Dual hole configurations

Based upon the above analysis, the medium sized area (9.62 mm^2^) was chosen to be split into two equal sized side-holes. The same tip volume used for the previous analysis was used to analyse the dual-hole configurations (Fig 3) and to generate the same parameters in Table 4.

**Table 4:** Tip volume parameters for the dual side-hole configurations.

Tip Design	Dual side-hole style	Inflow tip volume measurements			
		Max Velocity (m/s)	Average shear stress (Pa)	Percentage Shear stress >10 Pa	Total side-hole flow (mL/min)
Circular	Linear	1.77	3.67	8.58%	356
	Parallel	1.65	3.23	7.13%	363
Oval	Linear	1.84	4.15	10.1%	361
	Parallel	1.91	3.51	8.22%	376

**Fig 9:**
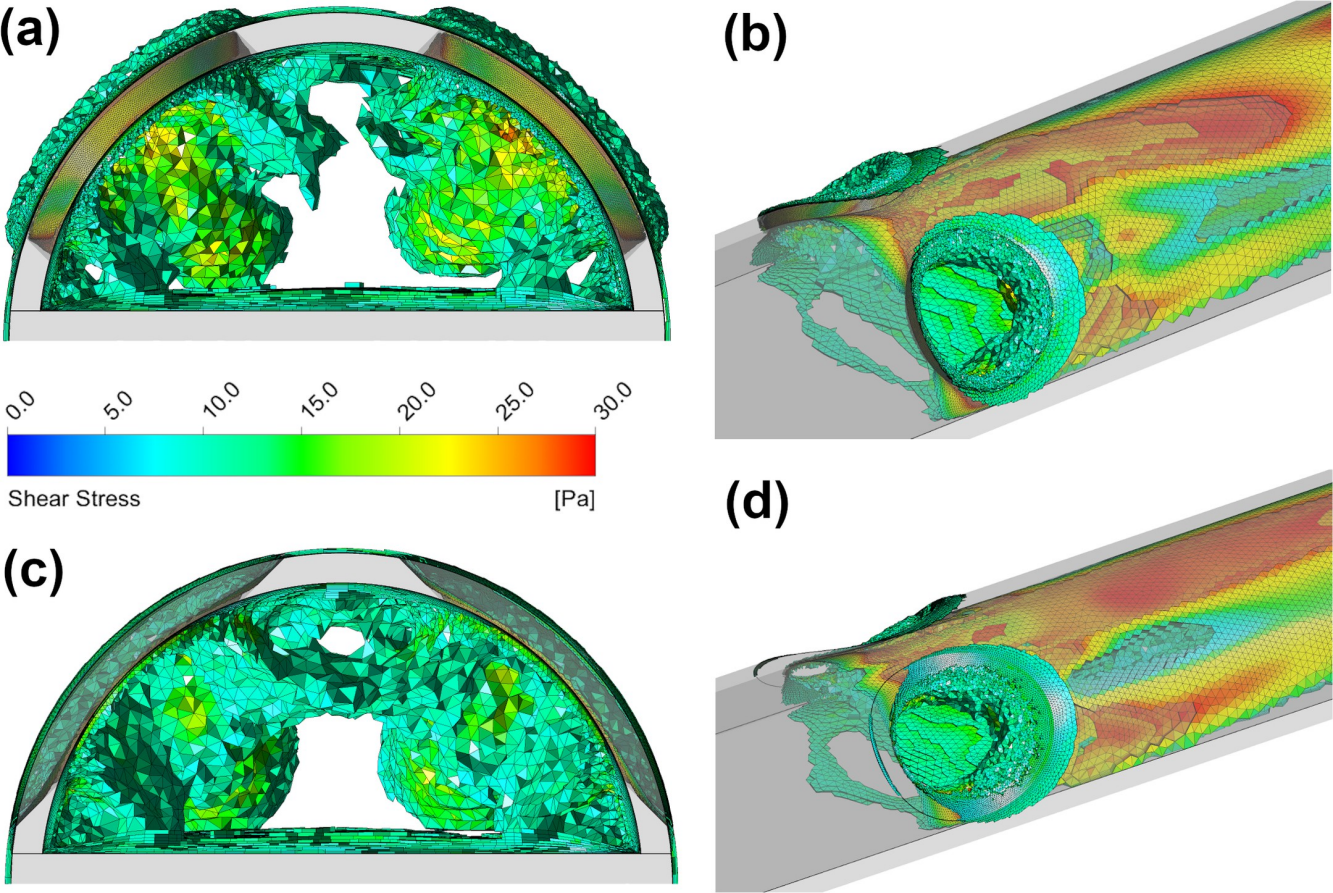
Regions inside the tip volume where shear stress >10 Pa for the dual-hole parallel circular (a)-(b) and oval (c)-(d) configurations.

As the linear configuration functions as two separate holes, the flow is split unevenly across each hole resulting in lower flow rates than the parallel configuration. This results in greater shear stress parameters as compared to the parallel configuration. Both dual-hole configurations lower the maximum velocity occurring in the tip when compared to the single medium hole with the same area (Table 2). However, the parallel configuration performs in a similar manner to the single medium-sized hole in shear parameters. As shown in Fig 9, there is a build-up of high shear stress around the exterior of the side-holes, which occludes a greater portion of the hole area given the reduction in size than a single side-hole.

The same platelet tracking model was also used to analyse the dual-hole configuration tips, with planes generated tangential to the surface of the catheter, 1 mm above each side-hole with the results tabulated in Table 5.

**Table 5:** Platelet parameters for the dual side-hole configurations.

Tip Design	Dual side-hole style	Inflow platelet model				
		Average residence time (s)	PLI	Average shear stress (Pa)	Percentage Shear stress >10 Pa	Average shear (s^-1^)
Circular	Linear	0.0126	0.0038	8.03	22.74	2219
	Parallel	0.0100	0.0089	7.63	17.03	2054
Oval	Linear	0.0159	0.0402	8.25	24.84	2282
	Parallel	0.0114	0.0108	8.48	23.47	2348

**Fig 10:**
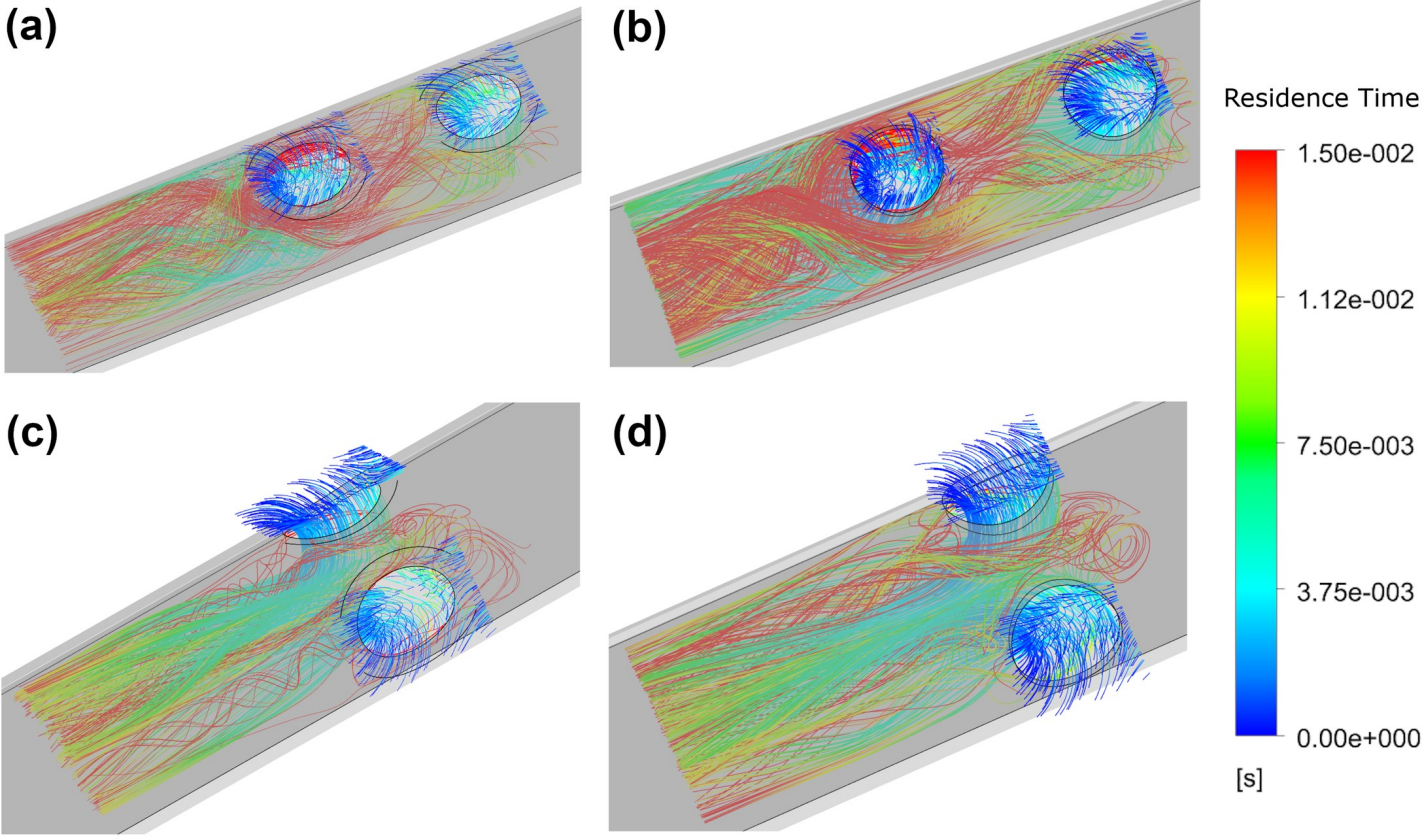
Residence times of inflowing platelets for the: Linear oval (a), Linear circular (b), Parallel oval (c) and Parallel circular (d).

The dual side-hole configurations experience similar residence times to the single hole tips, but with a much lower magnitude of PLI as a result of the reduction in high shear stress regions >10 Pa. The parallel arrangement has lower residence times than the linear configuration as the inflow is split equally, allowing for undisturbed inflow from both holes. In the linear configuration, the inflow from the second hole is obstructed as it flows down the lumen by the inflow from the first hole and can be seen in Fig 10.

**Fig 11:**
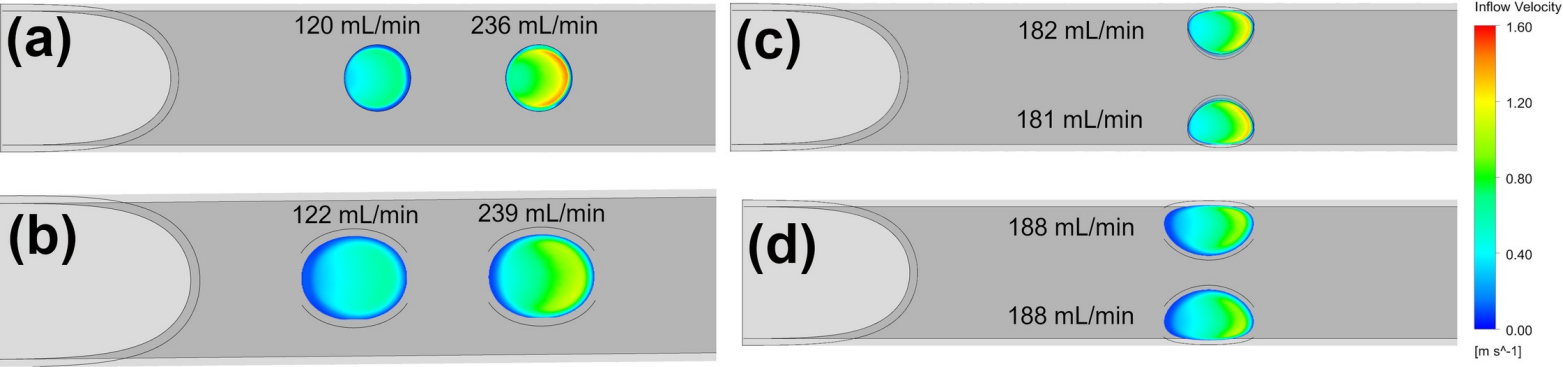
Velocity contours and flow rates for the Linear circular (a), Linear oval (b), Parallel circular (c) and Parallel oval (d).

Both linear configurations exhibit an uneven flow distribution, where the most proximal hole accounts for >66% of the total side-hole inflow, whereas the parallel configurations split the inflow evenly a shown in Fig 11. This low flow rate in the most distal side-hole results in more uniform low velocity distribution across the hole opening, whereas the side-holes which experience the higher flow rates of 180+ mL/min show regions of high velocity around the apex.

## Discussion

This study aimed to investigate the impact of different side-hole configurations on the local hemodynamics and performance of hemodialysis catheter tips. Simulations of multiple tip geometries were performed to critically assess the effects of side-hole geometry, area and number on the hemodynamics of inflowing blood on a simple symmetric tip representative of other designs currently in use. The inflow was assessed across a consistent volume from the tip of the catheter until the inflow was stable and normalised, comparing velocities, flow rates and regions of high shear stress. In addition, the path lines of platelets were also calculated to identify regions of high residence time and shear history which are combined in the platelet lysis index for each catheter tip design.

The inclusion of a side-hole in all the configurations tested improved the objective catheter performance (Table 1), with clear reductions in shear stress within the catheter lumen. The medium sized side-hole (9.62 mm^2^) performed the best, with increasing area decreasing the shear stress-based parameters as the larger area allowed for a more gradual redirection of inflow. However, if the side-hole became too large the inflow could ‘wash-out’ slowly through the tip (Figs 6D and 7B). It is unclear if this would be a benefit or drawback to function, as ‘wash-out’ could potentially be preventing platelet adhesion and thrombus formation as blood is kept moving, or alternatively, it may promote adhesion, as blood containing platelets activated by high-shear inflow through the side-hole are spending increased time around the catheter walls thereby increasing chances of adhesion instead of all side-hole inflow exiting the catheter. However, to date this has not been studied either *in vivo* or *in silico* and would require additional testing or a more sophisticated model capable of predicting clot or fibrin growth seeded in these regions.

Performance of the circular and oval-shaped side-holes was similar in most aspects with the largest difference being the larger regions where shear stress >10 Pa around the exterior of the circular holes. With this being used a threshold for platelet activation/blood damage, these areas would be the first to show signs of fibrin formation as shown by Lucas *et al*. [27] which match the regions shown in Figs 6 and 8. The higher average shear rate of the circular holes is resultant from the increased curvature, as well as the oval shape having an ‘angled-cut’ for a more gradual redirection of inflow indicating the oval shape as being more viable long term. With circular side-holes typically being drilled/punched [15], any imperfections/roughness will be ideal seed points for clotting/fibrin formation especially in the presence of high shear [28]. The use of laser cut side-holes as opposed to punched/drilled holes may be useful in reducing these seed points.

The best dual-hole configuration is clearly the parallel setup, with the lowest average shear stress, residence time and percentage shear stress <10 Pa. As the linear configuration concentrates the majority of inflow through the most proximal hole (Fig 11), the distal hole experiences a much lower flow rate, and if the catheter were to have additional holes, the flow is likely to become stagnant [29] allowing for increased residence time for platelets which should be avoided.

Increasing the number of side-holes may allow for the continued function of the catheter in the event one hole becomes occluded. However the reduction in side-hole area to achieve this results in a more significant portion having elevated shear stress <10 Pa, both on the exterior of the hole and inside the lumen. This larger region of elevated shear stress around the two much smaller holes implies the slow growth of fibrin formation [27, 30] may impact on these designs much quicker, as a greater proportion of each hole becomes blocked much faster than a single, larger side-hole.

Side-holes are included in many different styles of catheter tips, with associated benefits of increasing flow rates and spatial separation of inflow/outflow, hence reducing recirculation. Yet these benefits are offset by practical considerations; side-hole catheters have been prone to higher rates of infection and reduced effectiveness of heparin for clot removal and dysfunction from occluded side-holes [12, 28]. However the scale to which side-holes increases infection rates in the wide variety of designs on the market is not well understood, requiring a comprehensive *in vivo* study [12]. Side-holes should only be considered beneficial if any adverse effects on catheter patency are minimal enough to be offset by the increases in flow rate.

Whilst the methodology presented has been used extensively for analysing comparative catheter performance [10, 11, 27], a limitation of this study is the lack of accompanying *in vitro* or *in vivo* validation. Data on *in vitro* catheter performance is quite limited, with studies often using the same assumptions as CFD models of constant flow rate or an enclosed cylindrical SVC [13, 16, 29]. Whilst additional experimental testing is required, CFD studies can be used to highlight issues which *in vitro* studies can focus upon such as; Flow stagnation and increased residence times. An *in vitro* validation of these techniques was performed by Mareels *et al*. [16] and where possible the present study has reported similar parameters (Residence time, PLI, Average shear/stress) over similar ranges for easier comparisons. In previous comparative CFD catheter studies an overall PLI magnitude was calculated using the entrance velocity of the platelets as a weighting factor [10, 16], however, as this velocity is intrinsically linked to residence time, and is not included in the original Giersiepen *et al*. methodology [26], nor Goubergrits *et al*. implementation [31], the overall magnitude was taken as the average of the PLI magnitude summated for each platelet path line.

## Conclusion

The inclusion of side-holes on a symmetric tip catheter have been shown to increase the overall performance by increasing flow rates and decreasing shear stress. The side-hole size/shape requires careful consideration, with larger side-holes (>9.62 mm^2^) reducing shear stress for inflow, however, too large an area results in ‘wash-out’ which if unaccounted for could greatly increase the likelihood of platelet adhesion. An oval shaped hole appears preferable to a circular hole, as the gradual redirection of inflowing blood experiences lower average shear and has a decreased chance of hole occlusion via fibrin formation. The inclusion of multiple side-holes is best when both holes are parallel, yet the combination of two smaller side-holes is less desirable than a single, larger hole, as any occlusion of the smaller side-holes will impact performance more severely. Whilst side-holes may improve catheter performance, this should not come at the cost of reduced longevity *in vivo* and the determination of optimal design should be weighed against this. The ideal catheter would maximise the flow rate whilst minimising the chances of clotting/fibrin formation which can be achieved through optimising both the tip design and manufacturing processes.

## Supporting information

S1 File Values and calculations for the mesh independence study. (XLSX)

S1 Fig Centreline velocity contours for the (a) small circular, (b) small oval, (c) large circle, (d) circular linear, (e) oval linear, (f) circular parallel and (g) oval parallel side-hole configurations.
(TIF)

S2 Fig Residence time for the large oval shaped side-hole, with a legend showing the maximum residence time of 0.2 s. (TIF)
