# Supplementary figures and images for: Impact of side-hole geometry on the performance of hemodialysis catheter tips: A computational fluid dynamics assessment
Source: PLoS One. 2020 Aug 7;15(8):e0236946. doi: 10.1371/journal.pone.0236946 (PMC7413473; doi:10.1371/journal.pone.0236946)

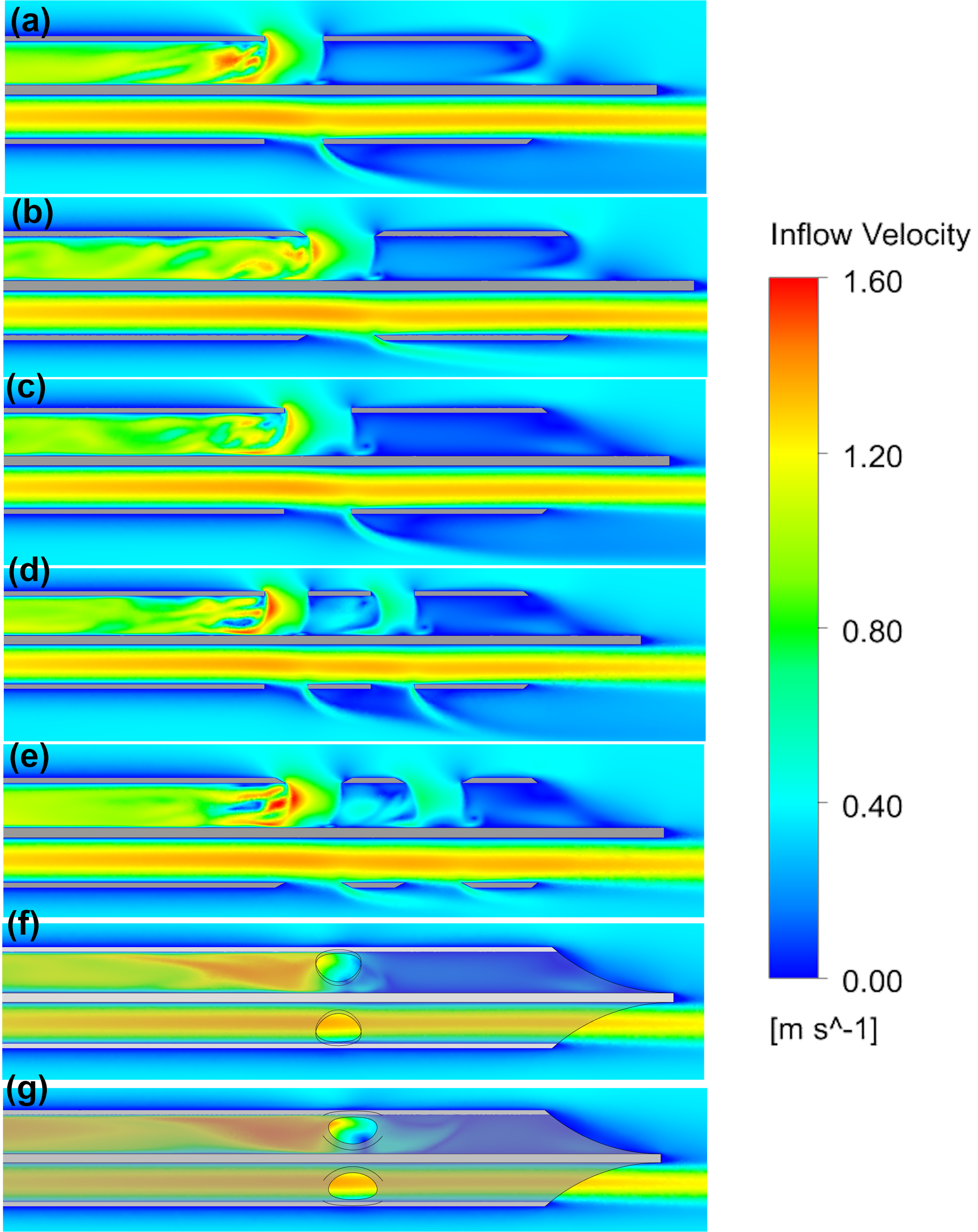

Supplement: S1 Fig — Centreline velocity contours for the (a) small circular, (b) small oval, (c) large circle, (d) circular linear, (e) oval linear, (f) circular parallel and (g) oval parallel side-hole configurations. (TIF) [file pone.0236946.s002.tif]

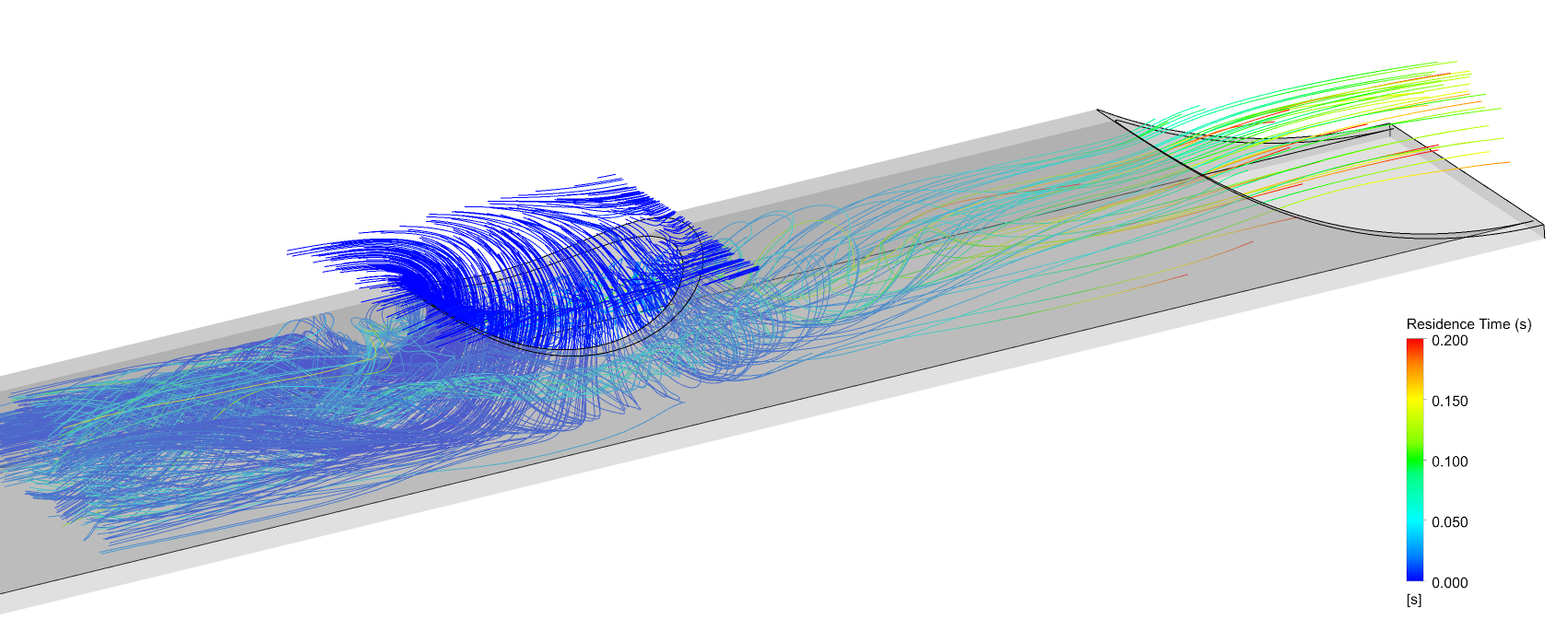

Supplement: S2 Fig — (TIF) [file pone.0236946.s003.tif]
